# Interactions between red and processed meat consumption and *APOA5* gene variants associated with the incidence of metabolic syndrome in Korean adults

**DOI:** 10.1186/s12263-022-00707-w

**Published:** 2022-04-25

**Authors:** Woo Jeong Choi, Dayeon Shin

**Affiliations:** Department of Food and Nutrition, Inha University, 100 Inha-ro, Michuhol-gu, Incheon, 22212 Republic of Korea

**Keywords:** *APOA5*, Metabolic syndrome, Single nucleotide polymorphism (SNP), Genome-wide association study (GWAS), Red and processed meat, Korean Genome and Epidemiology Study (KoGES)

## Abstract

**Background:**

Metabolic syndrome (MetS) is characterized by the coexistence of disorders such as diabetes, hypertension, hyperlipidemia, and obesity and is affected by genetic factors. Previous genome-wide association studies (GWAS) suggested that *APOA5* gene variants were significantly associated with MetS and its components. Dietary factors such as red and processed meat consumption can cause chronic diseases, including hypertension, diabetes, and vascular depression. The aim of this study was to investigate the modulation of the incidence of MetS by the interaction between *APOA5* rs662799 polymorphism and red and processed meat consumption.

**Methods:**

In this prospective cohort study, 3266 participants were collected from the Korea Association REsource (KARE) cohort of the Korean Genome and Epidemiology Study (KoGES) from 2001 to 2016. *APOA5* rs662799 polymorphism was extracted by GWAS using the Korean Chip. Red and processed meat consumption data were assessed using a semi-quantitative food frequency questionnaire.

**Results:**

The incidence of MetS in carriers of the minor G allele of rs662799 (AG + GG) and the third tertile of red and processed meat consumption (serving/day) was higher than those with the major allele of rs662799 (AA) and the first tertile of red and processed meat consumption (HR 1.70, 95% CI 1.30–2.22, *p* interaction = 0.002).

**Conclusions:**

An association between the presence of the minor alleles of rs662799 and high red and processed meat consumption and the incidence of MetS was observed in Korean adults.

**Supplementary Information:**

The online version contains supplementary material available at 10.1186/s12263-022-00707-w.

## Introduction

Metabolic syndrome (MetS) is characterized by disorders such as obesity, hyperlipidemia, hypertension, and diabetes [1] and is defined by the presence of three or more of the five components (high blood pressure, high waist circumference, high triglyceride, high high-density lipoprotein (HDL) cholesterol, and high fasting blood glucose) [2]. According to the Korean Statistical Information Service (KOSIS) 2019, the prevalence of MetS in middle-aged adults was 24.0% (21.8% women and 26.1% men) [3]. Previous meta-analyses have reported that MetS increases the risk of cardio-cerebrovascular disease, which is the primary cause of mortality increase [4, 5]. Hypertension, impaired fasting glucose, and obesity, which are used as criteria for MetS, are affected by genetic factors [6]. In various ethnic groups, results of previous genome-wide association studies (GWAS) suggested that the apolipoprotein A5 (*APOA5*) genetic variants were significantly associated with MetS and its five components [7–9].

*APOA5* gene encodes a protein that is a component of the lipoprotein fraction [10]. As the APOA5 protein acts as an activator of enzymes in lipid catabolism, genetic variants located on *APOA5* can enhance the metabolism of triglyceride-rich particles and affect triglyceride metabolism [11, 12]. A previous study investigated the relationship between variants in this gene region and an increased risk of coronary heart disease [13]. The rs662799 variant was significantly associated with plasma triglyceride levels, accompanied by a risk of heart disease [14].

Genetic and environmental factors, especially dietary factors, have a large effect on the risk of MetS [15, 16]. As the standard of living improves and dietary culture changes [1], meat consumption (including beef and pork) has increased considerably [17, 18]. Saturated fatty acids and cholesterol are more abundant in red meat than in white meat, such as poultry [19, 20]. Fat and cholesterol cause obesity and chronic diseases such as hypertension, diabetes, and vascular depression [21]. Compared to vegetarian diets, the consumption of red meat is associated with increased blood low-density lipoprotein (LDL) cholesterol and triglycerides [22]. Processed meat contains deleterious fats similar to that in red meat, and large quantities of salt (sodium) and nitrite are also present in processed meat [23, 24]. These constituents are known to be closely related to hypertension [23]. As symptoms such as hypertension, dyslipidemia, and obesity are components of MetS, the coexistence of these symptoms can affect the incidence of MetS [21, 25–27].

Studies investigating the interactions between red and processed meat consumption and *APOA5* rs662799 polymorphism in relation to the incidence of MetS using a prospective cohort study design are limited. We hypothesized that the incidence of MetS is modulated by the interaction between red and processed meat consumption and *APOA5* rs662799 polymorphism. Thus, the primary objective of this study was to investigate the modulation of the incidence of MetS based on the interaction between *APOA5* rs662799 polymorphism and red and processed meat consumption. Additionally, the interactions between red and processed meat consumption and *APOA5* rs662799 polymorphism in relation to the incidence of MetS components were assessed.

## Results

### General characteristics of the study population

Among the 3266 participants in this study, 1584 subjects developed MetS during the 16-year follow-up. The baseline characteristics of the participants according to the absence or presence of MetS (non-MetS and MetS, respectively) are shown in Table 1. The age of the participants in the MetS group was significantly higher than that in the non-MetS group. The population in Ansan was higher than that in Ansung, and the number of individuals who were educated over 7 years and under 12 years was higher than that with other education levels in both the non-MetS and MetS groups. Physical activity, body mass index (BMI), waist circumference, systolic blood pressure (SBP), diastolic blood pressure (DBP), fasting blood glucose (FBG), and triglyceride levels were significantly higher, and HDL cholesterol levels were significantly lower in the MetS group than in the non-MetS group (*p* < 0.05). In men, the proportion of current smokers was significantly higher than that of other smoking status groups for both non-MetS and MetS groups. BMI, waist circumference, SBP, DBP, FBG, and triglyceride levels were significantly higher, and HDL cholesterol levels were significantly lower in the MetS group than in the non-MetS group (*p* < 0.05). In women, the age of the MetS group was significantly older than those in the non-MetS group. Physical activity, BMI, waist circumference, SBP, DBP, FBG, and triglyceride levels were significantly higher, and HDL cholesterol level was significantly lower in the MetS group than in the non-MetS group (*p* < 0.05). Daily carbohydrate intake (g/day) was higher in the MetS group than in the non-MetS group.

**Table 1:** General characteristics of study participants at baseline according to the absence or presence of metabolic syndrome in Korean men and women

	Total (*n* = 3266)			Men (*n* = 1726)			Women (*n* = 1540)		
	Non-MetS (*n* = 1682)	MetS (*n* = 1584)	*p* value^1^	Non-MetS (*n* = 912)	MetS (*n* = 814)	*p* value^1^	Non-MetS (*n* = 770)	MetS (*n* = 770)	*p* value^1^
Age (years)	49.1 ± 8.1	50.8 ± 8.2	< 0.001	50.5 ± 8.5	50.2 ± 8.0	0.480	47.5 ± 7.2	51.5 ± 8.3	< 0.001
Residential area (*n*, %)			< 0.001			0.017			< 0.001
Ansan	521 (31.0)	733 (46.3)		308 (33.8)	320 (39.3)		213 (27.7)	413 (53.6)	
Ansung	1161 (69.0)	851 (53.7)		604 (66.2)	494 (60.7)		557 (72.3)	357 (46.4)	
Educational level (*n*, %)			< 0.001			0.618			< 0.001
≤ 6 years	323 (19.2)	428 (27.0)		142 (15.6)	125 (15.4)		181 (23.5)	303 (39.4)	
≤ 7 to ≤ 12 years	1049 (62.4)	924 (58.3)		541 (59.3)	500 (61.4)		508 (66.0)	424 (55.1)	
> 12 years	310 (18.4)	232 (14.6)		229 (25.1)	189 (23.2)		81 (10.5)	43 (5.6)	
Household income (*n*, %)			< 0.001			0.388			< 0.001
< 1 million	372 (22.1)	463 (29.2)		212 (23.2)	170 (20.9)		160 (20.8)	293 (38.1)	
≤ 1 to < 2 million	507 (30.1)	498 (31.4)		267 (29.3)	262 (32.2)		240 (31.2)	236 (30.6)	
≤ 2 to < 3 million	397 (23.6)	296 (18.7)		209 (22.9)	173 (21.3)		188 (24.4)	123 (16.0)	
≥ 3 million	406 (24.1)	327 (20.6)		224 (24.6)	209 (25.7)		182 (23.6)	118 (15.3)	
Smoking status (*n*, %)			0.042			0.001			0.313
Never	967 (57.5)	889 (56.1)		217 (23.8)	147 (18.1)		750 (97.4)	742 (96.4)	
Past	308 (18.3)	256 (16.2)		302 (33.1)	251 (30.8)		6 (0.8)	5 (0.6)	
Current	407 (24.2)	439 (27.7)		393 (43.1)	416 (51.1)		14 (1.8)	23 (3.0)	
Drinking status (*n*, %)			0.852			0.567			0.166
Never	698 (41.3)	642 (40.5)		176 (19.3)	142 (17.4)		519 (67.4)	500 (64.9)	
Past	92 (5.5)	92 (5.8)		78 (8.6)	67 (8.2)		14 (1.8)	25 (3.2)	
Current	895 (53.2)	850 (53.7)		658 (72.1)	605 (74.3)		237 (30.8)	245 (31.8)	
Physical activity (MET-h/day)	22.6 ± 13.7	24.3 ± 14.8	0.001	23.7 ± 14.3	24.5 ± 14.9	0.243	21.4 ± 12.8	24.0 ± 14.7	< 0.001
BMI (kg/m^2^)	22.9 ± 2.6	24.6 ± 2.6	< 0.001	22.8 ± 2.5	24.6 ± 2.5	< 0.001	23.1 ± 2.7	24.6 ± 2.8	< 0.001
Waist circumference (cm)	76.7 ± 7.2	82.1 ± 7.0	< 0.001	78.8 ± 6.2	84.2 ± 5.8	< 0.001	74.2 ± 7.6	80.0 ± 7.5	< 0.001
SBP (mmHg)	111.5 ± 14.2	119.2 ± 16.0	< 0.001	114.5 ± 13.8	121.0 ± 16.0	< 0.001	108.0 ± 13.8	117.3 ± 15.8	< 0.001
DBP (mmHg)	74.4 ± 9.7	79.7 ± 10.2	< 0.001	77.0 ± 9.5	81.8 ± 10.4	< 0.001	71.4 ± 9.0	77.4 ± 9.5	< 0.001
FBG (mg/dL)	82.6 ± 11.9	86.6 ± 17.6	< 0.001	85.0 ± 13.9	90.1 ± 20.6	< 0.001	79.7 ± 8.0	82.9 ± 12.7	< 0.001
Triglyceride (mg/dL)	116.6 ± 52.1	148.6 ± 95.6	< 0.001	126.6 ± 56.2	173.8 ± 114.2	< 0.001	104.7 ± 43.9	121.9 ± 60.5	< 0.001
HDL cholesterol (mg/dL)	49.5 ± 10.2	44.9 ± 9.0	< 0.001	48.0 ± 9.9	43.0 ± 8.6	< 0.001	51.2 ± 10.4	46.8 ± 9.0	< 0.001
Nutrient intake									
Energy (kcal/day)	1930.3 ± 559.5	1946.0 ± 585.6	0.434	1988.3 ± 544.0	2010.5 ± 554.0	0.400	1861.6 ± 570.2	1877.7 ± 610.3	0.594
Protein (g/day)	66.7 ± 24.3	66.6 ± 25.1	0.892	68.5 ± 23.9	69.9 ± 24.0	0.245	64.6 ± 24.7	63.1 ± 25.7	0.264
Fat (g/day)	34.0 ± 17.5	32.8 ± 17.9	0.050	35.9 ± 18.1	36.2 ± 17.6	0.755	31.8 ± 16.6	29.2 ± 17.5	0.003
Carbohydrate (g/day)	334.4 ± 92.7	341.1 ± 101.4	0.050	342.0 ± 88.4	345.7 ± 94.2	0.403	325.5 ± 96.8	336.3 ± 108.3	0.040
Fiber (g/day)	6.8 ± 3.1	7.0 ± 3.2	0.059	6.6 ± 2.9	6.9 ± 3.1	0.112	6.9 ± 3.24	7.1 ± 3.3	0.317

Data are presented as mean ± standard deviation or number (percentage, %)

*Abbreviations*: *MetS* Metabolic syndrome, *BMI* Body mass index, *SBP* Systolic blood pressure, *DBP* Diastolic blood pressure, *FBG* Fasting blood glucose, *HDL* High-density lipoprotein, *MET* Metabolic equivalent of task

^1^Chi-square test for categorical variables and *t*-test for continuous variables were performed to examine the differences between subjects with or without metabolic syndrome

### Association of genetic variants with metabolic syndrome and its components

GWAS analysis revealed eight single nucleotide polymorphisms (SNPs) that exhibited some degree of genome-wide significance (*p* < 5 × 10^−8^), suggesting that they were significantly associated with MetS after adjusting for age, sex, and residential area. The genetic model was based on an additive genetic model. Tables 2 and 3 show the results of the GWAS for *APOA5* variants (rs651821, rs662799, and rs2075291), *ZPR1* variants (rs75198898, rs113932726, and rs3741297), *BUD13* variant (rs74368849), and *FBXL17* variant (rs167012), which were associated with a significantly increased risk of MetS in the Korean population. Associations between each genetic variant and MetS components were analyzed using linear regression after adjusting for age, sex, and residential area (Tables 2 and 3). SBP and DBP were positively associated with the minor allele (risk allele) of the rs167012 variant (*p* < 0.05). FBG levels were was positively associated with six SNPs, except for rs651821 and rs662799 (*p* < 0.05). All eight SNPs showed a significantly negative association with HDL cholesterol and a positive association with triglyceride levels (*p* < 0.05).

**Table 2:** Results of significant association of genetic variants with metabolic syndrome and its components (waist circumference and systolic blood pressure) in Korean adults^a^

No.	SNP	Chr	Minor allele	MAF		Gene	Function	MetS (controls 5591; cases 2785)		Waist circumference		SBP	
				Cases	Controls			OR (95% CI)	Add *p* value	Beta ± SE	Add *p* value	Beta ± SE	Add *p* value
1	rs651821	11	C	0.350	0.278	*APOA5*	Intron	1.45 (1.32–1.58)	2.08 × 10^−15^	0.22 ± 0.17	0.201	0.53 ± 0.35	0.129
2	rs662799	11	G	0.349	0.278	*APOA5*	Intron	1.45 (1.32–1.58)	2.35 × 10^−15^	0.22 ± 0.17	0.211	0.53 ± 0.35	0.126
3	rs2075291	11	A	0.107	0.069	*APOA5*	Intron	1.70 (1.47–1.97)	1.42 × 10^−12^	0.43 ± 0.29	0.141	0.41 ± 0.58	0.480
4	rs75198898	11	A	0.107	0.069	*ZPR1*	Intron	1.69 (1.46–1.96)	2.25 × 10^−12^	0.38 ± 0.29	0.186	0.48 ± 0.58	0.404
5	rs113932726	11	T	0.107	0.069	*ZPR1*	Intron	1.69 (1.46–1.96)	2.25 × 10^−12^	0.38 ± 0.29	0.186	0.48 ± 0.58	0.404
6	rs3741297	11	T	0.107	0.070	*ZPR1*	Intron	1.69 (1.46–1.95)	2.97 × 10^−12^	0.41 ± 0.29	0.155	0.39 ± 0.58	0.498
7	rs74368849	11	A	0.107	0.071	*BUD13*	Intron	1.65 (1.42–1.90)	2.54 × 10^−11^	0.35 ± 0.29	0.221	0.39 ± 0.58	0.500
8	rs167012	5	C	0.421	0.366	*FBXL17*	Intron	1.29 (1.18–1.40)	1.52 × 10^−8^	0.31 ± 0.17	0.066	0.77 ± 0.33	0.020

*Abbreviations*: *SNP* Single nucleotide polymorphism, *Chr* Chromosome, *MAF* Minor allele frequency, *MetS* Metabolic syndrome, *OR* Odds ratio, *CI* Confidence interval, *SE* Standard error, *SBP* Systolic blood pressure, *DBP* Diastolic blood pressure, *FBG* Fasting blood glucose, *HDL* High-density lipoprotein, *Add* Additive model

^a^Models were adjusted for age, residential area, and sex

**Table 3:** Results of significant association of genetic variants with components of the metabolic syndrome (diastolic blood pressure, fasting blood glucose, HDL cholesterol, and triglyceride) in Korean adults^a^

No.	SNP	Chr	Minor allele	Gene	DBP		FBG		HDL cholesterol		Triglyceride	
					Beta ± SE	Add *p* value	Beta ± SE	Add *p* value	Beta ± SE	Add *p* value	Beta ± SE	Add *p* value
1	rs651821	11	C	*APOA5*	0.11 ± 0.23	0.617	0.55 ± 0.43	0.195	− 2.02 ± 0.20	1.08 × 10^−22^	33.44 ± 2.14	8.45 × 10^−54^
2	rs662799	11	G	*APOA5*	0.12 ± 0.23	0.612	0.54 ± 0.43	0.203	− 2.02 ± 0.20	8.70 × 10^−23^	33.44 ± 2.14	8.26 × 10^−54^
3	rs2075291	11	A	*APOA5*	0.31 ± 0.38	0.406	1.46 ± 0.71	0.040	− 3.37 ± 0.34	7.41 × 10^−23^	50.36 ± 3.58	2.81 × 10^−44^
4	rs75198898	11	A	*ZPR1*	0.37 ± 0.38	0.327	1.43 ± 0.71	0.044	− 3.34 ± 0.34	1.59 × 10^−22^	49.93 ± 3.57	1.23 × 10^−43^
5	rs113932726	11	T	*ZPR1*	0.37 ± 0.38	0.327	1.43 ± 0.71	0.044	− 3.34 ± 0.34	1.59 × 10^−22^	49.93 ± 3.57	1.23 × 10^−43^
6	rs3741297	11	T	*ZPR1*	0.27 ± 0.38	0.475	1.44 ± 0.71	0.042	− 3.36 ± 0.34	7.42 × 10^−23^	49.95 ± 3.57	1.03 × 10^−43^
7	rs74368849	11	A	*BUD13*	0.39 ± 0.38	0.303	1.41 ± 0.70	0.046	− 3.14 ± 0.34	2.79 × 10^−20^	47.40 ± 3.56	9.33 × 10^−40^
8	rs167012	5	C	*FBXL17*	0.44 ± 0.22	0.041	1.33 ± 0.41	0.001	− 0.44 ± 0.20	0.025	8.94 ± 2.08	1.79 × 10^−5^

*Abbreviations*: *SNP* Single nucleotide polymorphism, *Chr* Chromosome, *MAF* Minor allele frequency, *MetS* Metabolic syndrome, *OR* Odds ratio, *CI* Confidence interval, *SE* Standard error, *SBP* Systolic blood pressure, *DBP* Diastolic blood pressure, *FBG* Fasting blood glucose, *HDL* High-density lipoprotein, *Add* Additive model

^a^Models were adjusted for age, residential area, and sex

**Fig. 1:**
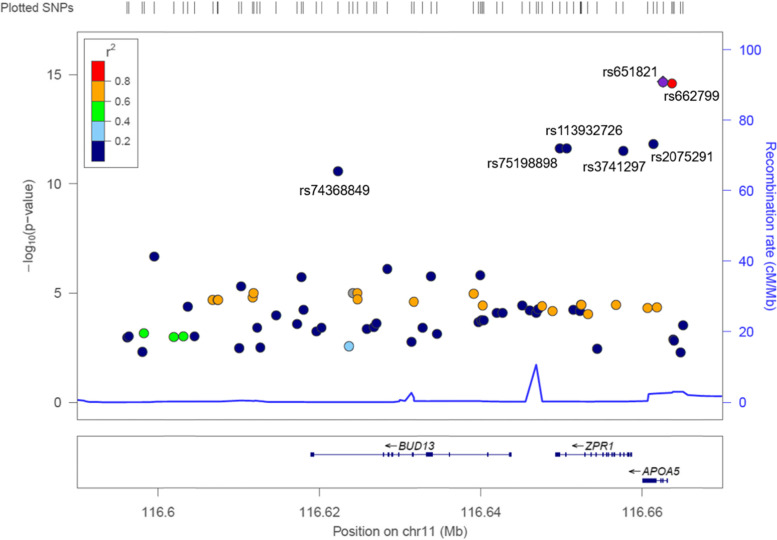
Regional plot for single nucleotide polymorphisms (SNPs) on *APOA5, ZPR1, and BUD13* genes that are significantly associated with metabolic syndrome (MetS). The SNPs shown in the figure are located on chromosome 11: 116.59–116.67 Mb, and numbered SNPs are the top seven SNPs associated with MetS. The SNPs are plotted by the statistical significance (-log10p value) of the associations. The blue line indicatess the recombination rates estimated using the 1000 Genomes November 2014 Asian population data. The purple diamond (rs651821) is the most significantly associated with MetS in Korean adults. The colors indicating the levels of linkage disequilibrium (r^2^) on the left side show the correlations between the purple diamond (rs651821) and other SNPs

Using a regional plot, 73 SNPs in the range from 116,590,000 to 116,670,000 on chromosome 11 were plotted, and the relationship between seven SNPs was confirmed (Fig. 1). Based on rs651821 with the lowest *p* value (2.08 × 10^−15^), the linkage disequilibrium (*r*^2^) of rs662799, which is also located in *APOA5*, was > 0.80, but the *r*^2^ of the other five SNPs (rs2075291, rs75198898, rs113932726, rs3741297, and rs74368849) was < 0.20. However, based on rs2075291, which had the lowest *p* value (1.42 × 10^−12^) among the five SNPs, the *r*^2^ between these five SNPs was > 0.80. The relationship group with *r*^2^ > 0.80 showed similar odds ratios (ORs) for MetS; ORs of rs651821 and rs662799 were 1.45 and 1.45, respectively and ORs of rs2075291, rs75198898, rs113932726, rs3741297, and rs74368849 were 1.70, 1.69, 1.69, 1.69, and 1.65. As the *FBXL17* rs167012 variant is located on chromosome 5, rs167012 was not plotted in Fig. 1.

### Interaction of *APOA5* rs662799 polymorphism and red and processed meat consumption in relation to the incidence of metabolic syndrome

Table 4 shows the adjusted hazard ratios (HRs) for the prospective associations between the *APOA5* rs662799 polymorphism and MetS incidence according to red and processed meat consumption (serving/day) in Koreans. After adjusting for age, residential area, education level, household income, smoking status, drinking status, physical activity, energy intake (kcal/day), white meat (g/day), fish (g/day), vegetable (g/day), fruit (g/day), and dairy (g/day), a significant association between red and processed meat consumption (serving/day) and *APOA5* rs662799 polymorphism and the incidence of MetS was observed in women. In women, compared to rs662799 AA genotype carriers in the first tertile group (reference), risk allele carriers (AG + GG) in the first tertile group (HR 1.47, 95% confidence interval (CI) 1.16–1.87), second tertile group (HR 1.48, 95% CI 1.15–1.90), and third tertile group (HR 1.70, 95% CI 1.30–2.22) had a significantly higher incidence of MetS (*p* interaction = 0.002). In men, significant associations between SNPs and the incidence of MetS by red and processed meat consumption (serving/day) were not observed. Similar to the interaction of red and processed meat consumption (serving/day), a significant association was observed in the interaction between red and processed meat consumption (g/day) and *APOA5* rs662799 and the incidence of MetS in women (Supplementary Table 1). Compared to rs662799 AA genotype carriers in the first tertile group in women, the adjusted HRs of risk allele carriers (AG + GG) in the first tertile (HR 1.37, 95% CI 1.08–1.73), second tertile (HR 1.44, 95% CI 1.13–1.85), and third tertile (HR 1.49, 95% CI 1.14–1.94) had a significantly higher incidence of MetS (*p* interaction = 0.009) (Supplementary Table 1).

**Table 4:** Interactions between red and processed meat consumption (serving/day) and *APOA5* rs662799 polymorphism with the incidence of metabolic syndrome in Korean adults

	Total (serving/day)			*p* interaction^1^	Men (serving/day)			*p* interaction^1^	Women (serving/day)			*p* interaction^1^
	Tertile 1	Tertile 2	Tertile 3		Tertile 1	Tertile 2	Tertile 3		Tertile 1	Tertile 2	Tertile 3	
Median	0.12	0.34	0.76		0.17	0.41	0.85		0.09	0.27	0.63	
Ranges	0.00–0.22	0.22–0.50	0.50–6.13		0.00–0.27	0.27–0.56	0.56–6.13		0.00–0.17	0.17–0.42	0.42–5.96	
Cases (*n*)/total (*n*)	541/1089	519/1088	524/1089		268/574	267/577	279/575		277/512	250/515	243/513	
rs662799												
AA	1.00 (reference)	1.11 (0.93–1.33)	1.20 (0.99–1.45)	< 0.001	1.00 (reference)	1.02 (0.79–1.33)	0.96 (0.73–1.27)	0.056	1.00 (reference)	1.38* (1.08–1.77)	1.16 (0.89–1.51)	0.002
AG + GG	1.36* (1.15–1.61)	1.47* (1.24–1.76)	1.42* (1.18–1.72)		1.27 (1.00–1.62)	1.21 (0.94–1.56)	1.24 (0.94–1.62)		1.47* (1.16–1.87)	1.48* (1.15–1.90)	1.70* (1.30–2.22)	

Data are presented as adjusted hazard ratios (HRs) and 95% confidence intervals (CIs). The total models were adjusted for residential area, age, sex, educational level, household income, drinking status, smoking status, physical activity, body mass index (BMI), energy intake (kcal/day), and intake of white meat (g/day), fish (g/day), vegetable (g/day), fruit (g/day), and dairy (g/day). The male and female models were adjusted for residential area, age, educational level, household income, drinking status, smoking status, physical activity, BMI, energy intake (kcal/day), and intake of white meat (g/day), fish (g/day), vegetable (g/day), fruit (g/day), and dairy (g/day)

^1^*p* interaction was obtained by genotype and red and processed meat consumption as categorical variables and adjusted for covariates

*p value < 0.05

### Interaction of *APOA5* rs662799 polymorphism and red and processed meat consumption in relation to the incidence of metabolic syndrome components

The associations between *APOA5* rs662799 polymorphism and red and processed meat consumption (serving/day) and the incidence of MetS components are presented in Table 5. In male carriers of the risk allele (AG + GG), the incidence of high triglyceride levels significantly increased across tertiles of red and processed meat consumption after multivariate adjustment (*p* interaction < 0.001). In women, carriers of the risk allele in the second tertile group (HR 1.28, 95% CI 1.05–1.56) and the third tertile group (HR 1.29, 95% CI 1.05–1.58) had a significantly higher incidence of low HDL cholesterol (*p* interaction = 0.001), compared to AA genotype carriers in the first tertile group (reference). In women, carriers of the risk allele had an increased incidence of high triglyceride levels (*p* interaction < 0.001), compared to the reference group. A significant association was also observed between the interaction of red and processed meat consumption (g/day) and *APOA5* rs662799 polymorphism in relation to the incidence of high triglyceride levels (Supplementary Table 2). The HR of high triglyceride levels in carriers of the risk allele (AG + GG) increased in both men (HRs 1.42–1.48, 95% CI 1.09–1.84, *p* interaction = 0.001) and women (HRs 1.41–1.75, 95% CI 1.09–2.24, *p* interaction < 0.001), compared to AA genotype carriers in the first tertile group; however, the HRs of the second tertile groups were the highest (Supplementary Table 2).

**Table 5:** Interactions between *APOA5* rs662799 polymorphism and red and processed meat consumption (serving/day) with the incidence of metabolic syndrome components in Korean adults

	Total (serving/day)			*p* interaction^1^	Men (serving/day)			*p* interaction^1^	Women (serving/day)			*p* interaction^1^
	Tertile 1	Tertile 2	Tertile 3		Tertile 1	Tertile 2	Tertile 3		Tertile 1	Tertile 2	Tertile 3	
Median	0.12	0.34	0.76		0.17	0.41	0.85		0.09	0.27	0.63	
Ranges	0.00–0.22	0.22–0.50	0.50–6.13		0.00–0.27	0.27–0.56	0.56–6.13		0.00–0.17	0.17–0.42	0.42–5.96	
Abdominal obesity												
Cases (*n*)/total (n)	643/1089	593/1088	568/1089		213/574	226/577	256/575		390/512	363/515	356/513	
AA	1.00 (reference)	1.12 (0.95–1.31)	1.27* (1.07–1.51)	0.171	1.00 (reference)	1.32* (1.01–1.73)	1.21 (0.91–1.62)	0.593	1.00 (reference)	1.12 (0.92–1.37)	1.15 (0.92–1.44)	0.228
AG + GG	1.06 (0.91–1.24)	1.11 (0.94–1.31)	1.17 (0.98–1.40)		1.11 (0.85–1.45)	0.95 (0.72–1.26)	1.13 (0.84–1.51)		1.05 (0.85–1.28)	1.08 (0.87–1.33)	1.19 (0.95–1.49)	
Elevated BP												
Cases (*n*)/total (*n*)	682/1089	628/1088	650/1089		397/574	372/577	388/575		300/512	264/515	239/513	
AA	1.00 (reference)	1.01 (0.87–1.18)	0.98 (0.83–1.15)	0.187	1.00 (reference)	0.95 (0.77–1.17)	0.82 (0.66–1.03)	0.181	1.00 (reference)	1.26 (1.00–1.58)	1.03 (0.80–1.32)	0.663
AG + GG	0.96 (0.83–1.12)	0.93 (0.80–1.09)	0.92 (0.78–1.09)		0.88 (0.72–1.07)	0.85 (0.69–1.05)	0.85 (0.68–1.06)		1.07 (0.85–1.34)	1.10 (0.86–1.41)	1.00 (0.77–1.30)	
Elevated FBG												
Cases (*n*)/total (*n*)	427/1089	467/1088	511/1089		290/574	303/577	318/575		165/512	163/515	166/513	
AA	1.00 (reference)	1.12 (0.93–1.36)	1.19 (0.98–1.45)	0.251	1.00 (reference)	1.01 (0.80–1.28)	0.94 (0.73–1.21)	0.653	1.00 (reference)	1.34 (1.00–1.81)	1.38* (1.01–1.90)	0.214
AG + GG	0.97 (0.81–1.18)	1.11 (0.92–1.34)	1.15 (0.94–1.40)		0.91 (0.72–1.15)	0.98 (0.77–1.24)	1.03 (0.80–1.32)		1.01 (0.74–1.38)	1.17 (0.85–1.61)	1.34 (0.96–1.87)	
Low HDL cholesterol												
Cases (*n*)/total (*n*)	796/1089	785/1088	737/1089		347/574	374/577	335/575		422/512	418/515	425/513	
AA	1.00 (reference)	1.09 (0.95–1.26)	0.93 (0.79–1.09)	< 0.001	1.00 (reference)	1.02 (0.81–1.27)	0.84 (0.65–1.07)	0.015	1.00 (reference)	0.98 (0.81–1.19)	1.02 (0.83–1.25)	0.001
AG + GG	1.26* (1.10–1.45)	1.32* (1.15–1.53)	1.30* (1.11–1.51)		1.17 (0.95–1.45)	1.35* (1.09–1.68)	1.16 (0.92–1.48)		1.20 (0.99–1.45)	1.28* (1.05–1.56)	1.29* (1.05–1.58)	
High triglyceride												
Cases (*n*)/total (*n*)	615/1089	626/1088	656/1089		369/574	379/577	402/575		265/512	241/515	241/513	
AA	1.00 (reference)	1.00 (0.84–1.19)	1.03 (0.85–1.24)	< 0.001	1.00 (reference)	1.01 (0.80–1.27)	1.24 (0.97–1.59)	< 0.001	1.00 (reference)	1.00 (0.77–1.31)	0.87 (0.65–1.16)	< 0.001
AG+GG	1.53* (1.30–1.81)	1.41* (1.19–1.67)	1.50* (1.25–1.80)		1.41* (1.13–1.75)	1.39* (1.11–1.74)	1.60* (1.26–2.02)		1.75* (1.36–2.24)	1.41* (1.09–1.83)	1.58* (1.20–2.07)	

Data are presented as adjusted hazard ratios (HRs) and 95% confidence intervals (CIs)

The total models were adjusted for residential area, age, sex, educational level, household income, drinking status, smoking status, physical activity, body mass index (BMI), energy intake (kcal/day), and intake of white meat (g/day), fish (g/day), vegetable (g/day), fruit (g/day), and dairy (g/day). The male and female models were adjusted for residential area, age, educational level, household income, drinking status, smoking status, physical activity, BMI, energy intake (kcal/day), and intake of white meat (g/day), fish (g/day), vegetable (g/day), fruit (g/day), and dairy (g/day)

*Abbreviations*: *BP* Blood pressure, *FBG* Fasting blood glucose, *HDL* High-density lipoprotein

^1^*p* interaction was obtained by genotype and red and processed meat consumption as categorical variables and adjusted for covariates

*p value < 0.05

## Discussion

In this prospective cohort study, the findings demonstrated that the interactions between red and processed meat consumption and *APOA5* rs662799 polymorphism were associated with the incidence of MetS and its components, such as high triglyceride and low HDL cholesterol levels in Korean adults.

A GWAS of MetS in Korean adults was performed using the Korean Chip (KCHIP) targeted for Korean adults, and significant GWAS findings regarding MetS were presented. After adjusting for age, sex, and residential area, the SNPs in *APOA5* were highly significantly associated withMetS and rs662799 was a tag SNP. Therefore, rs662799 was used for the analyses in this study.

The minor allele (G) of rs662799 *APOA5* increased the OR for MetS (OR 1.45, 95% CI 1.32–1.58) in Korean adults. Low HDL cholesterol (beta − 2.02, *p* = 8.70 × 10^−23^) and high triglyceride (beta 33.44, *p* = 8.26 × 10^−54^) levels were also associated with the G allele of rs662799. Similar to our findings in this study, the minor G allele of rs662799 increased the risk of MetS in Japanese (OR 1.57, 95% CI 1.29–1.90) and Hungarian (OR 3.62, 95% CI 1.20–10.94) populations [28, 29]. *APOA5* is known to affect triglyceride metabolism, and the G allele of rs662799 increases the risk of hypertriglyceridemia (OR 6.37, 95% CI 4.08–9.95) in the Taiwanese population [30]. In a northeast Chinese population, plasma triglyceride levels of the retained risk allele group of *APOA5* rs662799 polymorphism were significantly higher (2.66 mmol/L) than those in the non-retained group (1.90 mmol/L) (*p* = 0.01) [14]. Furthermore, carriers of the risk allele of rs662799 with MetS had higher plasma triglyceride levels (3.4 mmol/L) than non-carriers (2.9 mmol/L) (*p* = 0.001) in a case-control study of the Chinese population [11].

We hypothesized that the incidence of MetS is modulated by the interaction between *APOA5* rs662799 polymorphism and red and processed meat consumption. Through the analysis of interactions after adjusting for covariates, we found that women with the minor G allele of rs662799 (AG + GG) and in the third tertile of red and processed meat consumption (serving/day and g/day) had a higher incidence of MetS than those with the major allele of rs662799 (AA) and in the first tertile of processed and red meat consumption (HR 1.49–1.70, 95% CI 1.14–2.22). Furthermore, we found a significant interaction between rs662799 and processed and red meat consumption and the incidence of MetS components, such as low HDL cholesterol and high triglyceride levels. The interaction between the highest processed and red meat consumption and the minor G allele of *APOA5* rs662799 polymorphism was associated with the risk of high triglyceride levels (*p* interaction < 0.001) in men. In a previous follow-up study of adults over the age of 18, those with the A allele (AG + AA) of rs12970134 *MC4R* had an increased risk of hypertriglyceridemia in the highest consumption group of red meat (*p* < 0.05) [31]. The risk of central obesity was the highest in the AG + AA genotype group with the highest consumption of dietary fat (% energy) compared to the major allele (GG) carriers with the lowest consumption (*p* = 0.01) [31]. *APOA5* rs662799 polymorphism has also been studied for its interaction with genes and other environmental factors, except for dietary factors. The interaction between rs662799 and smoking habits significantly increased the risk of low HDL cholesterol and hypertriglyceridemia (*p* < 0.001) [32]. These studies suggest that dyslipidemia, such as hypertriglyceridemia and low HDL cholesterol, are MetS determination indices, and their significant interactions may affect the incidence of MetS. In Mexican populations aged 18 to 25 years, the risk of obesity (2.7 times, *p* = 0.006) and low HDL cholesterol (2.1 times, *p* = 0.018) was increased in those with the G allele of *APOA5* rs3135506 and saturated fatty acid intake ≥ 12 g/day [33].

Furthermore, saturated fatty acids and cholesterol contained in red and processed meat affect the risk of developing chronic diseases such as hypertension, vascular depression, and obesity [21]. Compared with vegetarians, the red meat consumption group had increased levels of LDL cholesterol and triglycerides [22]. A prospective cohort study by Kim and Shin [25] reported that the relative risk of hyper-LDL cholesterolemia was 1.23 times higher (95% CI 1.08–1.41, *p* = 0.0021), and the relative risk of hypercholesteremia was 1.15 times higher (95% CI 1.04–1.28, *p* = 0.0082) in men with a 60-g increase in red meat intake. In women, the relative risk of hypercholesterolemia was 1.08, with a 60-g increase in red meat intake (95% CI 1.02–1.15, *p* = 0.0145) [25].

The results of the interaction analyses showed different effects according to sex in the present study. Immoderate abdominal fat, obesity, and weight gain associated with adipose depots can increase the risk of metabolic complications [34]. In the Korean population, the risk of MetS in those who had low dietary fat consumption was increased by high dietary carbohydrate consumption, showing that other dietary factors can cause disease [35]. Additionally, a previous study reported that excessive low-fat consumption could be a reason for the incidence of MetS [36]. Several studies suggest that sex differences in the absorption and metabolism of lipids and other nutrients contribute to the difference in metabolic disease risk between men and women [37, 38]. There is increasing resarch into sex differences in metabolism, and understanding of how metabolic pathways are controlled and coordinated is needed.

The present study had several limitations and strengths. First, the findings may not be applicable to other populations or races, because this study was conducted in a Korean population. The second limitation is that the semiquantitative food frequency questionnaire (SQ-FFQ) used for the analysis was based only on the data recorded at baseline. The dietary habits of participants could change depending on the situation or time. Despite these limitations, this study had several strengths. To the best of our knowledge, this is the first study to investigate the interaction between red and processed meat consumption and *APOA5* rs662799 polymorphism on the incidence of MetS in Korean adults. Second, the present study was conducted while controlling for various potential covariates such as dietary factors, demographic characteristics, and anthropometric measurements. Lastly, this was a prospective cohort study to examine the cause-and-effect relationship between the consumption of red and processed meat and *APOA5* rs662799 polymorphism in relation to the incidence of MetS.

## Conclusion

Interaction analyses between *APOA5* rs662799 polymorphism and red and processed meat consumption revealed a significant association between the presence of the minor G allele of rs662799 and high consumption of red and processed meat and the incidence of MetS in Korean adults. The incidence of MetS was increased by 70% in women who were AG + GG carriers and belonged to the high red and processed meat consumption group after adjustment for potential covariates (*p* interaction < 0.05). The present study provides a new understanding of the association between genetic factors, dietary factors, and MetS. These findings will be useful to establish guidelines for targeted management, based on individual genetic information, to prevent the incidence of MetS and its components.

## Materials and methods

### Study population

This prospective cohort study used data collected from the Korea Association REsource (KARE) cohort of the Korea Genome and Epidemiology Study (KoGES) from 2001–2002 (baseline examination) to 2015–2016 (7th follow-up examination) (Ansan and Ansung areas). The KoGES-Ansan and Ansung study targeted individuals over 40 years of age living in urban (Ansan) and rural (Ansung) cities. All participants submitted written informed consent at the baseline interviews and were surveyed for biochemical data, anthropometric data, medical history, demographic data, and dietary intake at healthcare institutions [39].

This study used KoGES-Ansan and Ansung data from the years 2001–2016 (*n* = 10,030). Among the Ansan and Ansung cohort populations, DNA samples from 5493 participants were genotyped at baseline. Participants with no genetic information (*n* = 4537) or a history of cardiovascular disease, stroke, or cancer were excluded (*n* = 172). In summary, 5321 participants were included in the GWAS analysis.

**Fig. 2:**
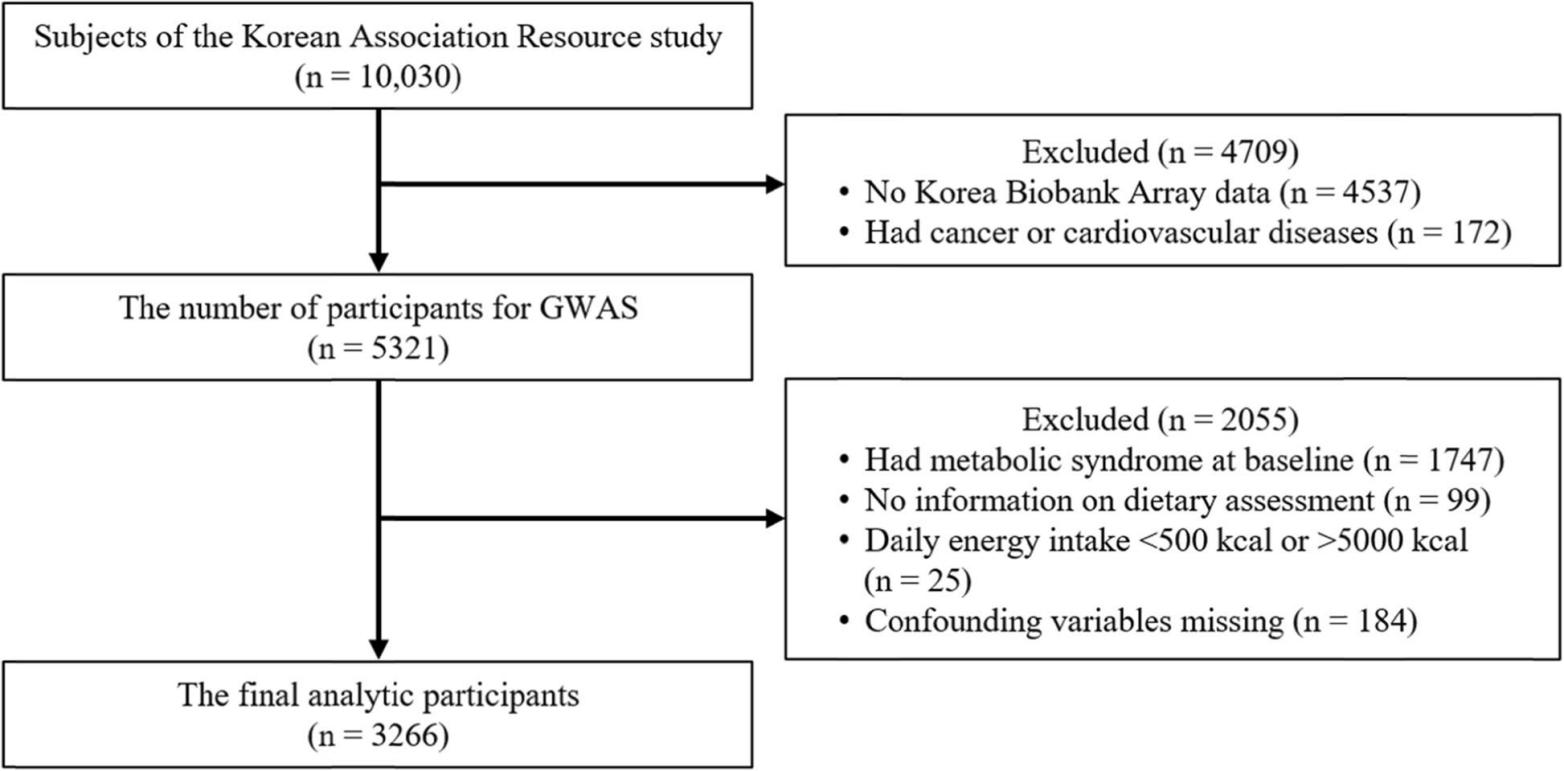
Flowchart of the study population

To examine the associations between red and processed meat consumption and the incidence of MetS, participants who had MetS at baseline (*n* = 1747) were excluded. Participants who did not complete the baseline questionnaire for red and processed meat consumption (*n* = 1099), those who reported implausible energy intake of < 500 kcal/day or > 5000 kcal/day (*n* = 25), and those who did not have information on covariates or five criteria data of MetS at baseline (*n* = 184) were also excluded. A total of 3266 participants (1726 men and 1540 women) were included in the association analysis (Fig. 2). The study protocol was reviewed and approved by the Institutional Review Board (IRB) of Inha University on January 31, 2020 (IRB No. 200129-1A).

### General characteristics, anthropometric measurements, and biochemical variables

Demographic characteristics, including age (years), sex, residential area (Ansan and Ansung), educational level (≤ 6 years, ≤ 7 to ≤ 12 years, and > 12 years), household income (< 1 million, ≤ 1 to < 2 million, and ≤ 2 to < 3 million), smoking status (never, past, and current), drinking status (never, past, and current), and physical activity (metabolic equivalent of task-hour/day; MET-h/day) were collected using an interviewer-administered questionnaire. Waist circumference (cm), height (cm), and weight (kg) were measured in light clothing without shoes, and BMI was calculated as weight (kg) divided by height squared (m^2^). Blood pressure (mmHg) was measured after 5 min of rest in a seated position. SBP was measured at Korotkoff phase I (the first time when a repetitive cardiac impulse sound appeared), and DBP was measured at Korotkoff phase V (the time when repetitive cardiac impulse sound disappeared). SBP and DBP were used as the averages of the left- and right-arm blood pressures. Blood samples were collected after 8 h of overnight fasting. Biochemical marker analyses, including FBG, triglyceride, and HDL cholesterol, were performed using an ADVIA 1650 chemistry analyzer (Siemens, New York, NY, USA).

### Definition of metabolic syndrome

MetS was defined according to the NCEP ATP III guidelines [2]. MetS was defined as the presence of three or more of the following five characteristics: (1) SBP ≥ 130 mmHg, DBP ≥ 85 mmHg, taking an antihypertensive agent, having a history of hypertension diagnosis, or treatment for hypertension; (2) serum triglyceride level ≥ 150 mg/dL; (3) FBG ≥ 100 mg/dL, using insulin injection, taking oral hypoglycemic agent, or having a history of a diabetes diagnosis; (4) serum HDL cholesterol level < 40 mg/dL in men and < 50 mg/dL in women; and (5) waist circumference ≥ 90 cm for men and ≥ 80 cm for women. Participants with MetS at baseline were excluded, and those who acquired new-onset MetS during the follow-up period were considered to have MetS. These five parameters were defined as MetS components.

### Dietary measurements

Dietary intake data were assessed using a validated 106 item semi-quantitative food frequency questionnaire (SQ-FFQ). Dietary intake data were evaluated based on the SQ-FFQ at baseline for participants who acquired MetS between the baseline and the 7th follow-up survey.

Daily nutrient intake data included carbohydrate (g/day), fat (g/day), protein (g/day), fiber (g/day), and energy (kcal/day) intake. Among the 106 items recorded in the SQ-FFQ, red meat comprised braised pork, roasted pork, pork belly, roasted beef, beefsteak, and edible viscera, and processed meat comprising sausage and ham. For additional covariate information, white meat (fried chicken and chicken stew, g/day), fish (tuna/canned tuna, dried anchovy, octopus/dried octopus, Alaska pollack, flat fish, sea bream, yellow croaker, eel, hair tail, Spanish mackerel, Pacific saury, mackerel, and sushi; g/day), vegetable (black mushroom, button mushroom, oyster mushroom, stem of taro/sweet potato, bracken, bean sprouts, doraji/deoduck, perilla leaf, lettuce, spinach, Korean cabbages, radish/salted radish, zucchini, pumpkin, green pepper, carrot/carrot juice, cucumber, water dropwort, leek, crown daisy, *Aster scaber, pimpinella brachycarpa*, and pepper leaves; g/day), fruit (tomato/cherry tomato, grape, orange, apple, pear, tangerine, persimmon/dried persimmon, banana, plum, peach, watermelon, and strawberry; g/day), and dairy (milk and yogurt) were assessed using the SQ-FFQ.

The SQ-FFQ was recorded based on participants’ frequency and portion size of dietary intake during the follow-up period. The questionnaire on the frequency of dietary intake was divided into nine responses for each food (rarely or never, one time/month, two or three times/month, one or two times/week, three or four times/week, five or six times/week, one time/day, two times/day, and three times or more/day). The questionnaire on portion size was divided into three responses for each food item (half-serving, one-serving, and two or more servings). For the analysis, red and processed meat consumption (serving/day) was calculated based on the reported portion size of each food. Red and processed meat consumption (serving/day and g/day) was classified into three groups (tertiles 1, 2, and 3).

### Genotyping and imputation

DNA samples from the participants were extracted from peripheral blood samples. The imputed genotypes were produced by the Korea Biobank Array (Korean Chip, KCHIP, Seoul, South Korea) for research on genetic factors of diseases in the Korean population [40]. KCHIP data were provided by the Center for Genomic Science, Korea National Institute of Health (4845-301, 3000-3001), and the total number of single-nucleotide variants included in the KCHIP array was 833,535. According to standard quality control procedures, markers with a missing rate > 0.05, minor allele frequency < 0.01, and *p-*value of Hardy-Weinberg equilibrium (HWE) < 1.0 × 10^−6^ were excluded.

After genotyping and sample quality control, GWAS was conducted to extract SNPs significantly associated with MetS in KoGES-Ansan and Ansung subjects adjusted for age, sex, and residential area (Bonferroni *p* value < 5.0 × 10^−8^). A total of eight SNPs (rs651821, rs662799, and rs2075291 in *APOA5*; rs75198898, rs113932726, and rs3741297 in *ZPR1*; rs74368849 in *BUD13*; and rs167012 in *FBXL17*) were extracted by GWAS analysis. The SNPs in *APOA5* were the most significantly associated with MetS in Korean adults, and rs662799 was confirmed to be a tag SNP. Consequently, *APOA5*rs662799 polymorphism was selected for the analysis in this study.

### Statistical analyses

GWAS anlysis for selecting SNPs associated with MetS and its components was performed using the PLINK software (version 1.90 beta, https://www.cog-genomics.org/plink/1.9). The association of SNPs with MetS was based on an additive genetic model and analyzed using logistic regression analysis, adjusting for age, sex, and residential area as covariates. The association between SNPs and MetS components (waist circumference, blood pressure, FBG, HDL cholesterol, and triglyceride levels) was analyzed using linear regression analysis after adjusting for age, sex, and residential area. To obtain regional association plots, the web-based program Locuszoom version 1.3 (http://csg.sph.umich.edu/locuszoom/) was used.

All analyses were conducted separately for men and women, and participants were categorized into three groups according to dietary consumption: red and processed meat (tertile 1, tertile 2, and tertile 3). Continuous variables (age, physical activity, BMI, waist circumference, SBP, DBP, FBG, HDL cholesterol, triglyceride, and daily intake of energy, protein, fat, carbohydrate, and fiber) for general characteristics were expressed as mean and standard deviation, and categorical variables (sex, residential area, educational level, household income, smoking status, and drinking status) were expressed as numbers and percentages. Differences between the MetS group and non-MetS group were examined using the *t*-test for continuous variables and the chi-square test for categorical variables. The HRs and 95% CIs for the incidence of MetS and its components according to the interaction between red and processed meat consumption and *APOA5* rs662799 polymorphism were estimated using multivariable Cox proportional hazards models. The analyses were conducted after adjusting for residential area, age, sex, educational level, household income, drinking status, smoking status, physical activity, BMI, energy intake (kcal/day), and intake of white meat (g/day), fish (g/day), vegetable (g/day), fruit (g/day), and dairy (g/day) as covariates. All statistical analyses were performed using Statistical Package for the Social Sciences (SPSS) software (version 25.0; SPSS Inc., IBM, New York, NY, USA). Statistical significance was considered at a two-sided *p*-value < 0.05.

## Supplementary Information

**Additional file 1: Supplementary Table 1.** Interactions between red and processed meat consumption (g/day) and *APOA5* rs662799 polymorphism on the incidence of metabolic syndrome in Korean adults. **Supplementary Table 2.** Interactions between red and processed meat consumption (g/day) and *APOA5* rs662799 polymorphism on the incidence of metabolic syndrome components in Korean adults.

## Data Availability

The dataset used in this study (Ansan-Ansung Cohort Study of the KoGES) can be provided after the review and evaluation of the research plan by the Korea National Institute of Health, Korea Disease Control and Prevention Agency (http://nih.go.kr/contents.es?mid=a50401010400).

## Abbreviations

*APOA5*: Apolipoprotein A5
MetS: Metabolic syndrome
SNP: Single nucleotide polymorphism
GWAS: Genome-wide association study
HDL: High-density lipoprotein
KoGES: Korea Genome Epidemiology Study
KARE: Korea Association REsource
SQ-FFQ: Semi-quantitative food frequency questionnaire
KCHIP: Korean Chip
OR: Odds ratio
HR: Hazard ratio
CI: Confidence interval
BMI: Body mass index
IRB: Institutional Review Board
MET: Metabolic equivalent of task
SBP: Systolic blood pressure
DBP: Diastolic blood pressure
FBG: Fasting blood glucose
HWE: Hardy-Weinberg equilibrium
SPSS: Statistical Package for the Social Sciences
