# Supplementary material for: Interactions between red and processed meat consumption and APOA5 gene variants associated with the incidence of metabolic syndrome in Korean adults
Source: Genes Nutr. 2022 Apr 25;17:5. doi: 10.1186/s12263-022-00707-w (PMC9040260; doi:10.1186/s12263-022-00707-w)
Supplement: Supplementary file 1 — Additional file 1: Supplementary Table 1. Interactions between red and processed meat consumption (g/day) and APOA5 rs662799 polymorphism on the incidence of metabolic syndrome in Korean adults. Supplementary Table 2. Interactions between red and processed meat consumption (g/day) and APOA5 rs662799 polymorphism on the incidence of metabolic syndrome components in Korean adults. [file 12263_2022_707_MOESM1_ESM.docx]

**Title:** Interactions between Red and Processed Meat Consumption and *APOA5* Gene Variants Associated with the Incidence of Metabolic Syndrome in Korean Adults

**Authors:** Woo Jeong Choi, Dayeon Shin*

**Affiliation:**

Department of Food and Nutrition, Inha University, 100 Inha-ro, Michuhol-gu, Incheon 22212, Republic of Korea.

***Corresponding Author:**

Dayeon Shin, PhD, RD

Assistant Professor

Department of Food and Nutrition

Inha University

100 Inha-ro, Michuhol-gu, Incheon 22212

Republic of Korea

Office: +82-32-860-8123

E-mail: [dyshin@inha.ac.kr](mailto:dyshin@inha.ac.kr)

Supplementary Table 1 Interactions between red and processed meat consumption (g/day) and *APOA5* rs662799 polymorphism on the incidence of metabolic syndrome in Korean adults.

|  | Total (g/day) | | | *p*  Interaction^1^ | Men (g/day) | | | *p*  Interaction^1^ | | Women (g/day) | | | *p*  Interaction^1^ | |
| --- | --- | --- | --- | --- | --- | --- | --- | --- | --- | --- | --- | --- | --- | --- |
|  | Tertile 1 | Tertile 2 | Tertile 3 |  | Tertile 1 | Tertile 2 | Tertile 3 | |  | Tertile 1 | Tertile 2 | Tertile 3 | |  |
| Median | 12.50 | 35.48 | 80.95 |  | 17.50 | 43.81 | 89.94 | |  | 9.00 | 28.48 | 66.88 | |  |
| Ranges | 0.00 - 23.04 | 23.33 - 53.18 | 53.21 - 591.96 |  | 0.00 - 29.17 | 29.25 - 64.29 | 64.35 - 591.96 | |  | 0.00 - 17.50 | 17.92 - 41.81 | 42.08 - 468.04 | |  |
| Cases (n) / Total (n) | 546 / 1083 | 530 / 1093 | 508 / 1090 |  | 271 / 578 | 270 / 573 | 273 / 575 | |  | 281 / 514 | 257 / 512 | 232 / 514 | |  |
| rs662799 |  |  |  | 0.001 |  |  |  | | 0.240 |  |  |  | | 0.009 |
| AA | 1.00 (reference) | 1.08 (0.91 - 1.29) | 0.99 (0.82 - 1.20) |  | 1.00 (reference) | 1.02 (0.79 - 1.32) | 0.89 (0.68 - 1.17) | |  | 1.00 (reference) | 1.18 (0.92 - 1.50) | 1.07 (0.82 - 1.39) | |  |
| AG + GG | 1.28* (1.08 - 1.52) | 1.37* (1.15 - 1.63) | 1.31* (1.09 - 1.58) |  | 1.26 (0.99 - 1.61) | 1.26 (0.98 - 1.61) | 1.10 (0.84 - 1.44) | |  | 1.37* (1.08 - 1.73) | 1.44* (1.13 - 1.85) | 1.49* (1.14 - 1.94) | |  |

Data are presented as adjusted hazard ratios (HRs) and 95% confidence intervals (CIs). Abbreviations: BP, blood pressure; FBG, fasting blood glucose; HDL, high-density lipoprotein. The total models were adjusted for residential area, age, sex, educational level, household income, drinking status, smoking status, physical activity, body mass index (BMI), energy intake (kcal/day), and intake of white meat (g/day), fish (g/day), vegetable (g/day), fruit (g/day), and dairy (g/day). The male and female models were adjusted for residential area, age, educational level, household income, drinking status, smoking status, physical activity, BMI, energy intake (kcal/day), and intake of white meat (g/day), fish (g/day), vegetable (g/day), fruit (g/day), and dairy (g/day). ^1^*p* Interaction was obtained by genotype and red and processed meat consumption as categorical variables and adjusted for covariates. **p* Value <0.05.

Supplementary Table 2 Interactions between red and processed meat consumption (g/day) and *APOA5* rs662799 polymorphisms on the incidence of metabolic syndrome components in Korean adults.

|  | Total (g/day) | | | *p*  Interaction^1^ | Men (g/day) | | | *p*  Interaction^1^ | Women (g/day) | | | *p*  Interaction^1^ |
| --- | --- | --- | --- | --- | --- | --- | --- | --- | --- | --- | --- | --- |
|  | Tertile 1 | Tertile 2 | Tertile 3 |  | Tertile 1 | Tertile 2 | Tertile 3 |  | Tertile 1 | Tertile 2 | Tertile 3 |  |
| Median | 12.50 | 35.48 | 80.95 |  | 17.50 | 43.81 | 89.94 |  | 9.00 | 28.48 | 66.88 |  |
| Ranges | 0.00 - 23.04 | 23.33 - 53.18 | 53.21 - 591.96 |  | 0.00 - 29.17 | 29.25 - 64.29 | 64.35 - 591.96 |  | 0.00 - 17.50 | 17.92 - 41.81 | 42.08 - 468.04 |  |
| Abdominal obesity |  |  |  |  |  |  |  |  |  |  |  |  |
| Cases (n) / Total (n) | 641 / 1083 | 597 / 1093 | 566 / 1090 |  | 216 / 578 | 226 / 573 | 253 / 575 |  | 389 / 514 | 364 / 512 | 356 / 514 |  |
| AA | 1.00 (reference) | 1.15 (0.98 - 1.35) | 1.30* (1.09 - 1.54) | 0.116 | 1.00 (reference) | 1.29 (0.99 - 1.70) | 1.12 (0.84 - 1.49) | 0.285 | 1.00 (reference) | 0.97 (0.79 - 1.19) | 1.20 (0.96 - 1.49) | 0.095 |
| AG + GG | 1.06 (0.91 - 1.25) | 1.14 (0.97 - 1.35) | 1.20* (1.00 - 1.43) |  | 1.08 (0.83 - 1.42) | 1.00 (0.75 - 1.32) | 1.00 (0.74 - 1.34) |  | 0.94 (0.77 - 1.16) | 1.11 (0.91 - 1.37) | 1.15 (0.92 - 1.44) |  |
| Elevated BP |  |  |  |  |  |  |  |  |  |  |  |  |
| Cases (n) / Total (n) | 666 / 1083 | 642 / 1093 | 652 / 1090 |  | 388 / 578 | 374 / 573 | 395 / 575 |  | 303 / 514 | 267 / 512 | 233 / 514 |  |
| AA | 1.00 (reference) | 1.08 (0.92 - 1.26) | 0.93 (0.74 - 1.09) | 0.456 | 1.00 (reference) | 1.07 (0.87 - 1.31) | 0.93 (0.75 - 1.16) | 0.666 | 1.00 (reference) | 1.26* (1.00 - 1.58) | 1.01 (0.79 - 1.31) | 0.663 |
| AG + GG | 0.92 (0.79 - 1.07) | 0.94 (0.80 - 1.10) | 0.97 (0.82 - 1.14) |  | 0.90 (0.73 - 1.10) | 0.93 (0.75 - 1.14) | 0.97 (0.78 - 1.21) |  | 1.09 (0.87 - 1.37) | 1.02 (0.80 - 1.30) | 1.04 (0.80 - 1.36) |  |
| Elevated FBG |  |  |  |  |  |  |  |  |  |  |  |  |
| Cases (n) / Total (n) | 432 / 1083 | 467 / 1093 | 506 / 1090 |  | 294 / 578 | 303 / 573 | 314 / 575 |  | 165 / 514 | 169 / 512 | 160 / 514 |  |
| AA | 1.00 (reference) | 1.05 (0.87 - 1.26) | 1.05 (0.86 - 1.28) | 0.706 | 1.00 (reference) | 0.97 (0.77 - 1.23) | 0.90 (0.71 - 1.16) | 0.939 | 1.00 (reference) | 1.19 (0.88 - 1.60) | 1.34 (0.98 - 1.84) | 0.367 |
| AG + GG | 0.96 (0.79 - 1.16) | 1.01 (0.83 - 1.22) | 1.05 (0.86 - 1.28) |  | 0.91 (0.72 - 1.15) | 0.99 (0.78 - 1.24) | 0.95 (0.74 - 1.21) |  | 0.93 (0.68 - 1.28) | 1.28 (0.94 - 1.74) | 1.14 (0.81 - 1.60) |  |
| Low HDL-cholesterol |  |  |  |  |  |  |  |  |  |  |  |  |
| Cases (n) / Total (n) | 802 / 1083 | 774 / 1093 | 742 / 1090 |  | 354 / 578 | 365 / 573 | 334 / 575 |  | 427 / 514 | 416 / 512 | 422 / 514 |  |
| AA | 1.00 (reference) | 0.95 (0.82 - 1.09) | 0.92 (0.78 - 1.08) | <0.001 | 1.00 (reference) | 0.90 (0.72 - 1.13) | 0.78 (0.61 - 1.00) | 0.030 | 1.00 (reference) | 0.96 (0.79 - 1.16) | 0.95 (0.77 - 1.17) | 0.012 |
| AG + GG | 1.19* (1.03 - 1.36) | 1.25* (1.08 - 1.44) | 1.25* (1.07 - 1.46) |  | 1.12 (0.91 - 1.38) | 1.24* (1.00 - 1.53) | 1.09 (0.87 - 1.38) |  | 1.20 (0.99 - 1.46) | 1.27* (1.04 - 1.54) | 1.19 (0.97 - 1.46) |  |
| High triglyceride |  |  |  |  |  |  |  |  |  |  |  |  |
| Cases (n) / Total (n) | 605 / 1083 | 631 / 1093 | 661 / 1090 |  | 372 / 578 | 389 / 573 | 389 / 575 |  | 269 / 514 | 246 / 512 | 232 / 514 |  |
| AA | 1.00 (reference) | 1.05 (0.88 - 1.25) | 0.97 (0.81 - 1.18) | <0.001 | 1.00 (reference) | 1.08 (0.86 - 1.36) | 1.05 (0.82 - 1.35) | 0.001 | 1.00 (reference) | 0.92 (0.71 - 1.20) | 0.75 (0.56 - 1.00) | 0.002 |
| AG+GG | 1.57* (1.33 - 1.86) | 1.36* (1.15 - 1.61) | 1.51* (1.27 - 1.81) |  | 1.48* (1.19 - 1.84) | 1.36* (1.09 - 1.70) | 1.42* (1.12 - 1.80) |  | 1.64* (1.28 - 2.10) | 1.36* (1.04 - 1.78) | 1.41* (1.09 - 1.82) |  |

Data are presented as adjusted hazard ratios (HRs) and 95% confidence intervals (CIs). Abbreviations: BP, blood pressure; FBG, fasting blood glucose; HDL, high-density lipoprotein. The total models were adjusted for residential area, age, sex, educational level, household income, drinking status, smoking status, physical activity, body mass index (BMI), energy intake (kcal/day), and intake of white meat (g/day), fish (g/day), vegetable (g/day), fruit (g/day), and dairy (g/day). The male and female models were adjusted for residential area, age, educational level, household income, drinking status, smoking status, physical activity, BMI, energy intake (kcal/day), and intake of white meat (g/day), fish (g/day), vegetable (g/day), fruit (g/day), and dairy (g/day). ^1^*p* Interaction was obtained by genotype and red and processed meat consumption as categorical variables and adjusted for covariates. **p* Value <0.05.
